# Crystal structure of 4-chloro-*N*-[2-(piperidin-1-yl)eth­yl]benzamide monohydrate

**DOI:** 10.1107/S2056989014026851

**Published:** 2015-01-01

**Authors:** K. Prathebha, D. Reuben Jonathan, B. K Revathi, S. Sathya, G. Usha

**Affiliations:** a PG and Research Department of Physics, Queen Mary’s College, Chennai-4, Tamilnadu, India; b Department of Chemistry, Madras Christian College, Chennai-59, India

**Keywords:** crystal structure, piperidine, benzamide, monohydrate, hydrogen bonding

## Abstract

In the title compound, C_14_H_19_ClN_2_O_2_·H_2_O, the piperdine ring adopts a chair conformation. The dihedral angle between the mean plane of the piperidine ring and that of the phenyl ring is 41.64 (1)°. In the crystal, mol­ecules are linked by O—H⋯N, N—H⋯O and C—H⋯O hydrogen bonds involving the water mol­ecule, forming double-stranded chains propagating along [010].

## Related literature   

For the synthesis of the title compound, see: Prathebha *et al.* (2013 ▸, 2014 ▸). For the biological activities of piperdine derivatives, see: Pandey & Chawla (2012 ▸); Jayalakshmi & Nanjundan (2008 ▸); Parthiban *et al.* (2005 ▸); Aridoss *et al.* (2008 ▸); Ramachandran *et al.* (2011 ▸). For related structures, see: Prathebha *et al.* (2014 ▸); Ávila *et al.* (2010 ▸); Al-abbasi *et al.* (2010 ▸).
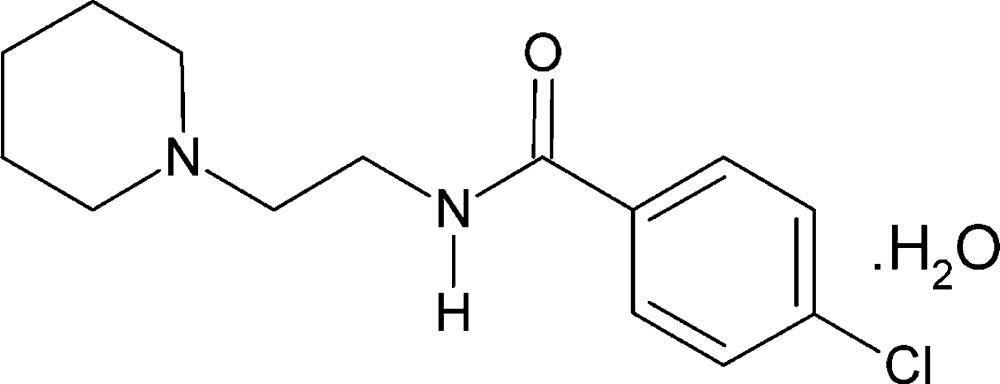


## Experimental   

### Crystal data  

C_14_H_19_ClN_2_O·H_2_O
*M* *_r_* = 284.78
Monoclinic, 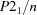

*a* = 14.9115 (6) Å
*b* = 6.6899 (3) Å
*c* = 15.6215 (7) Å
β = 102.956 (2)°
*V* = 1518.67 (11) Å^3^
*Z* = 4
Mo *K*α radiation
μ = 0.25 mm^−1^
*T* = 293 K
0.25 × 0.23 × 0.20 mm

### Data collection  

Bruker Kappa APEXII CCD diffractometer
Absorption correction: multi-scan (*SADABS*; Bruker, 2004 ▸) *T*_min_ = 0.939, *T*_max_ = 0.951
12566 measured reflections
3780 independent reflections
1953 reflections with *I* > 2σ(*I*)
*R*_int_ = 0.036

### Refinement  

*R*[*F* ^2^ > 2σ(*F* ^2^)] = 0.051
*wR*(*F* ^2^) = 0.160
*S* = 1.01
3780 reflections
181 parameters
2 restraints
H atoms treated by a mixture of independent and constrained refinement
Δρ_max_ = 0.28 e Å^−3^
Δρ_min_ = −0.21 e Å^−3^

### 

Data collection: *APEX2* (Bruker, 2004 ▸); cell refinement: *SAINT* (Bruker, 2004 ▸); data reduction: *XPREP* in *SAINT*; program(s) used to solve structure: *SHELXS97* (Sheldrick, 2008 ▸); program(s) used to refine structure: *SHELXL97* (Sheldrick, 2008 ▸); molecular graphics: *ORTEP-3 for Windows* (Farrugia, 2012 ▸); software used to prepare material for publication: *SHELXL97*, *PLATON* (Spek, 2009 ▸) and *publCIF* (Westrip, 2010 ▸).

## Supplementary Material

Crystal structure: contains datablock(s) I, New_Global_Publ_Block. DOI: 10.1107/S2056989014026851/su5034sup1.cif

Structure factors: contains datablock(s) I. DOI: 10.1107/S2056989014026851/su5034Isup2.hkl

Supporting information file. DOI: 10.1107/S2056989014026851/su5034Isup3.cml

**Fig. 1.**
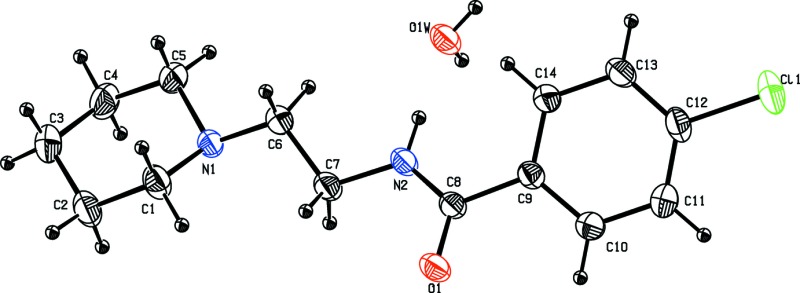
The molecular structure of the title compound, showing the atom labelling. Displacement ellipsoids are drawn at the 30% probability level.

**Fig. 2.**
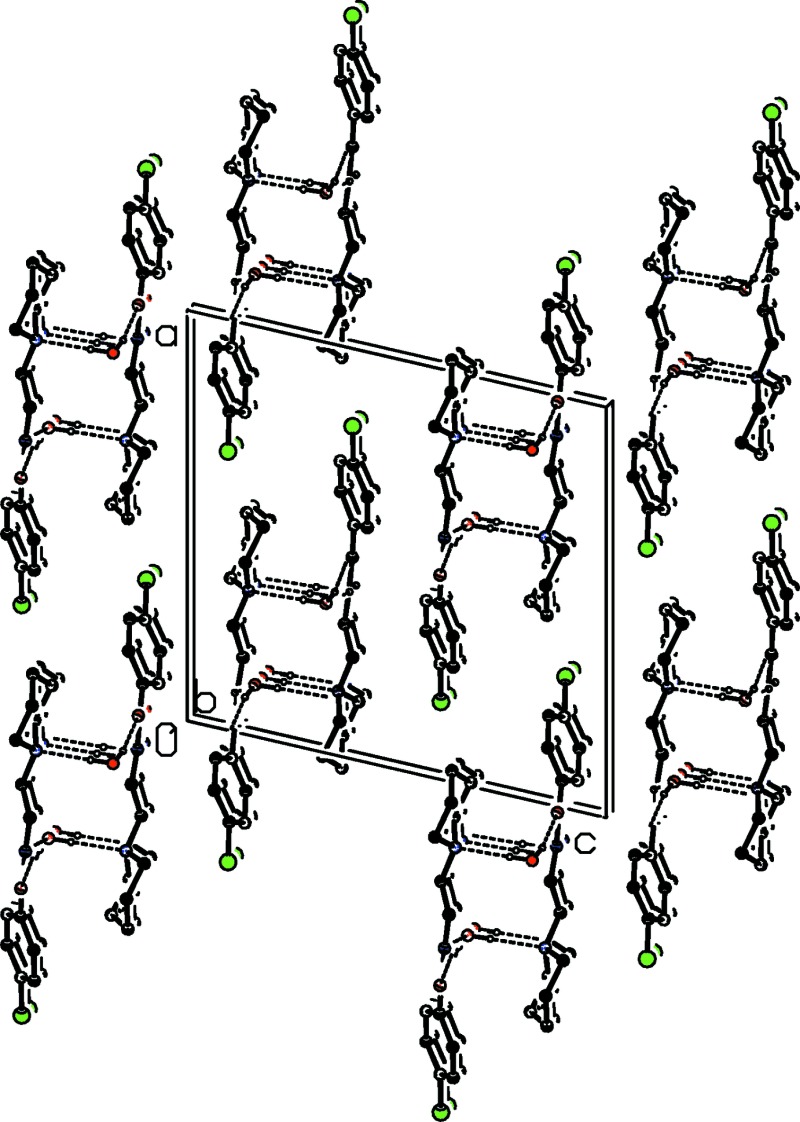
A view along the b axis of the crystal packing of the title compound. The dashed lines indicate the hydrogen bonds (see Table 1 for details; C-bound H atoms have been omitted for clarity).

. DOI: 10.1107/S2056989014026851/su5034fig1.tif
The mol­ecular structure of the title compound, showing the atom labelling. Displacement ellipsoids are drawn at the 30% probability level.

b . DOI: 10.1107/S2056989014026851/su5034fig2.tif
A view along the *b* axis of the crystal packing of the title compound. The dashed lines indicate the hydrogen bonds (see Table 1 for details; C-bound H atoms have been omitted for clarity).

CCDC reference: 1038084

Additional supporting information:  crystallographic information; 3D view; checkCIF report

## Figures and Tables

**Table 1:** Hydrogen-bond geometry (, )

*D*H*A*	*D*H	H*A*	*D* *A*	*D*H*A*
O1*W*H1*WA*N1^i^	0.83(2)	2.03(2)	2.851(3)	174(2)
N2H2O1*W*	0.86	2.06	2.855(2)	153
C6H6*B*O1*W*	0.97	2.59	3.406(3)	142

Symmetry code: (i) 
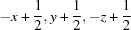
.

## supplementary crystallographic information

### S1. Comment

The piperidine derivatives were reported to have antimicrobial activity. Piperidine derivatives have been synthesized for pharmaceutical research as they are very efficient against resistance microorganisms. The substituted piperidine derivatives were also reported to have antimicrobial activity (Pandey & Chawla, 2012; Jayalakshmi & Nanjundan, 2008; Parthiban *et al.*, 2005; Aridoss *et al.*, 2008; Ramachandran *et al.* 2011).

In the title compound, Fig. 1, the piperidine ring is *cis* to the phenyl ring. The C—N distances [1.335 (2) - 1.464 (2) Å] are in the normal range and are in good agreement with values of from similar structures (Ávila *et al.*, 2010; Prathebha *et al.*, 2014). The bond angle sum around atoms N1 and N2 [333.2 (4)° and 359.97 (1)°, respectively] shows sp^3^ hybridization. The C═O distance [1.231 (2) Å] is comparable with the value reported previously (Al-abbasi *et al.*, 2010). The piperdine ring adopts a chair conformation with puckering parameters of q2 = 0.6994 (0) Å, φ2 = 88.60 (0)° q3 = -0.0267 (0) Å, QT = 0.6999 Å and θ2 = 92.19 (2)°.

In the crystal, adjacent molecules are linked by O-H···N, O-H···O and C-H···O hydrogen bonds, involving the water molecule, forming double stranded chains propagating along [010]; see Table 1 and Fig. 2

### S2. Experimental

The title compound was synthesized following a publish procedure (Prathebha *et al.*, 2013, 2014). In a 250 mL round-bottomed flask 120 mL of ethylmethylketone was added to 1,2-aminoethylpiperidine (0.02 mol) and stirred at room temperature. After 5 min triethylamine (0.04 mol) was added and the mixture was stirred for 15 min. Then 4-chlorobenzoylchloride (0.04 mol) was added and the reaction mixture was stirred at room temperature for ca. 2 h. A white precipitate of triethylammoniumchloride was formed. It was filtered and the filtrate was evaporated to give the crude product. It was recrystallized twice from ethylmethylketone (yield: 82%) giving colourless block-like crystals of the title compound.

### S3. Refinement

The water H atoms were located in a difference Fourier map and freely refined. The NH and C-bound H atoms were positioned geometrically and treated as riding on their parent atoms: C—H = 0.93 - 0.97 Å, N—H = 0.86 Å with *U*_iso_(H) = 1.5*U*_eq_(C) for methyl H atoms and = 1.2U_eq_(N,C) for other H atoms.

### Crystal data

**Table:**

C_14_H_19_ClN_2_O·H_2_O	*F*(000) = 608
*M**_r_* = 284.78	*D*_x_ = 1.245 Mg m^−^^3^
Monoclinic, *P*2_1_/*n*	Mo *K*α radiation, λ = 0.71073 Å
Hall symbol: -P 2yn	Cell parameters from 3780 reflections
*a* = 14.9115 (6) Å	θ = 1.7–28.4°
*b* = 6.6899 (3) Å	µ = 0.25 mm^−^^1^
*c* = 15.6215 (7) Å	*T* = 293 K
β = 102.956 (2)°	Block, colourless
*V* = 1518.67 (11) Å^3^	0.25 × 0.23 × 0.20 mm
*Z* = 4	

### Data collection

**Table:**

Bruker Kappa APEXII CCD diffractometer	3780 independent reflections
Radiation source: fine-focus sealed tube	1953 reflections with *I* > 2σ(*I*)
Graphite monochromator	*R*_int_ = 0.036
ω and φ scan	θ_max_ = 28.4°, θ_min_ = 1.7°
Absorption correction: multi-scan (*SADABS*; Bruker, 2004)	*h* = −19→19
*T*_min_ = 0.939, *T*_max_ = 0.951	*k* = −8→7
12566 measured reflections	*l* = −20→20

### Refinement

**Table:**

Refinement on *F*^2^	Primary atom site location: structure-invariant direct methods
Least-squares matrix: full	Secondary atom site location: difference Fourier map
*R*[*F*^2^ > 2σ(*F*^2^)] = 0.051	Hydrogen site location: inferred from neighbouring sites
*wR*(*F*^2^) = 0.160	H atoms treated by a mixture of independent and constrained refinement
*S* = 1.01	*w* = 1/[σ^2^(*F*_o_^2^) + (0.0714*P*)^2^ + 0.2535*P*] where *P* = (*F*_o_^2^ + 2*F*_c_^2^)/3
3780 reflections	(Δ/σ)_max_ < 0.001
181 parameters	Δρ_max_ = 0.28 e Å^−^^3^
2 restraints	Δρ_min_ = −0.21 e Å^−^^3^

### Special details

**Table:**

Geometry. All e.s.d.'s (except the e.s.d. in the dihedral angle between two l.s. planes) are estimated using the full covariance matrix. The cell e.s.d.'s are taken into account individually in the estimation of e.s.d.'s in distances, angles and torsion angles; correlations between e.s.d.'s in cell parameters are only used when they are defined by crystal symmetry. An approximate (isotropic) treatment of cell e.s.d.'s is used for estimating e.s.d.'s involving l.s. planes.
Refinement. Refinement of *F*^2^ against ALL reflections. The weighted *R*-factor *wR* and goodness of fit *S* are based on *F*^2^, conventional *R*-factors *R* are based on *F*, with *F* set to zero for negative *F*^2^. The threshold expression of *F*^2^ > σ(*F*^2^) is used only for calculating *R*-factors(gt) *etc*. and is not relevant to the choice of reflections for refinement. *R*-factors based on *F*^2^ are statistically about twice as large as those based on *F*, and *R*- factors based on ALL data will be even larger.

### Fractional atomic coordinates and isotropic or equivalent isotropic displacement parameters (Å^2^)

**Table:**

	*x*	*y*	*z*	*U*_iso_*/*U*_eq_	
O1W	0.36430 (12)	−0.0064 (2)	0.33083 (13)	0.0544 (4)	
H1WA	0.3601 (17)	−0.005 (4)	0.2772 (11)	0.062 (8)*	
H1WB	0.3987 (15)	0.082 (3)	0.3533 (15)	0.064 (8)*	
C1	0.13069 (15)	−0.6539 (4)	0.39904 (16)	0.0561 (6)	
H1A	0.1347	−0.6240	0.4605	0.067*	
H1B	0.1773	−0.7528	0.3957	0.067*	
C2	0.03637 (15)	−0.7388 (4)	0.35887 (18)	0.0671 (7)	
H2A	0.0342	−0.7795	0.2988	0.081*	
H2B	0.0257	−0.8563	0.3916	0.081*	
C3	−0.03859 (16)	−0.5866 (4)	0.35964 (18)	0.0682 (7)	
H3A	−0.0428	−0.5605	0.4197	0.082*	
H3B	−0.0973	−0.6390	0.3279	0.082*	
C4	−0.01743 (15)	−0.3941 (4)	0.31710 (18)	0.0665 (7)	
H4A	−0.0223	−0.4163	0.2549	0.080*	
H4B	−0.0621	−0.2930	0.3234	0.080*	
C5	0.07842 (15)	−0.3203 (4)	0.35883 (17)	0.0610 (7)	
H5A	0.0910	−0.1994	0.3293	0.073*	
H5B	0.0817	−0.2878	0.4200	0.073*	
C6	0.24037 (14)	−0.3914 (3)	0.38842 (15)	0.0508 (6)	
H6A	0.2500	−0.3780	0.4517	0.061*	
H6B	0.2448	−0.2593	0.3641	0.061*	
C7	0.31449 (13)	−0.5225 (3)	0.36732 (16)	0.0511 (6)	
H7A	0.3226	−0.6393	0.4051	0.061*	
H7B	0.2961	−0.5678	0.3069	0.061*	
C8	0.48147 (13)	−0.5074 (3)	0.38619 (13)	0.0411 (5)	
C9	0.56372 (13)	−0.3766 (3)	0.39205 (13)	0.0405 (5)	
C10	0.63653 (15)	−0.4479 (4)	0.35953 (15)	0.0530 (6)	
H10	0.6343	−0.5769	0.3370	0.064*	
C11	0.71251 (15)	−0.3293 (4)	0.36017 (16)	0.0633 (7)	
H11	0.7610	−0.3773	0.3377	0.076*	
C12	0.71562 (14)	−0.1406 (4)	0.39423 (16)	0.0552 (6)	
C13	0.64543 (15)	−0.0669 (4)	0.42879 (15)	0.0541 (6)	
H13	0.6491	0.0607	0.4529	0.065*	
C14	0.56919 (14)	−0.1860 (3)	0.42700 (14)	0.0479 (5)	
H14	0.5209	−0.1371	0.4497	0.058*	
N1	0.14836 (11)	−0.4716 (2)	0.35340 (11)	0.0435 (4)	
N2	0.40087 (11)	−0.4147 (3)	0.37969 (11)	0.0480 (5)	
H2	0.4002	−0.2864	0.3830	0.058*	
O1	0.48880 (10)	−0.6908 (2)	0.38625 (10)	0.0573 (4)	
Cl1	0.81091 (5)	0.01012 (12)	0.39483 (6)	0.0952 (3)	

### Atomic displacement parameters (Å^2^)

**Table:**

	*U*^11^	*U*^22^	*U*^33^	*U*^12^	*U*^13^	*U*^23^
O1W	0.0567 (10)	0.0380 (10)	0.0677 (12)	−0.0102 (8)	0.0123 (9)	0.0000 (9)
C1	0.0484 (13)	0.0450 (14)	0.0746 (15)	−0.0061 (11)	0.0129 (11)	0.0122 (12)
C2	0.0503 (14)	0.0549 (16)	0.0971 (19)	−0.0147 (12)	0.0184 (13)	0.0019 (14)
C3	0.0445 (13)	0.0754 (19)	0.0887 (18)	−0.0116 (13)	0.0234 (12)	−0.0014 (15)
C4	0.0427 (13)	0.0672 (18)	0.0939 (18)	0.0093 (12)	0.0244 (12)	0.0050 (15)
C5	0.0478 (13)	0.0479 (15)	0.0937 (18)	0.0037 (11)	0.0297 (12)	−0.0020 (13)
C6	0.0437 (12)	0.0424 (13)	0.0682 (14)	−0.0064 (10)	0.0164 (10)	−0.0050 (11)
C7	0.0364 (11)	0.0373 (13)	0.0779 (15)	−0.0021 (9)	0.0091 (10)	−0.0042 (11)
C8	0.0383 (11)	0.0353 (12)	0.0482 (12)	−0.0031 (9)	0.0067 (8)	−0.0020 (9)
C9	0.0365 (10)	0.0375 (12)	0.0456 (11)	−0.0029 (9)	0.0049 (8)	0.0003 (9)
C10	0.0440 (12)	0.0439 (13)	0.0720 (15)	−0.0020 (10)	0.0150 (11)	−0.0097 (11)
C11	0.0442 (13)	0.0648 (18)	0.0857 (18)	−0.0042 (12)	0.0247 (12)	−0.0076 (14)
C12	0.0413 (12)	0.0530 (16)	0.0710 (15)	−0.0140 (10)	0.0121 (11)	0.0048 (12)
C13	0.0495 (13)	0.0394 (13)	0.0718 (15)	−0.0083 (10)	0.0102 (11)	−0.0057 (11)
C14	0.0397 (11)	0.0420 (13)	0.0628 (14)	−0.0029 (9)	0.0129 (10)	−0.0057 (11)
N1	0.0361 (9)	0.0344 (10)	0.0610 (11)	−0.0010 (7)	0.0132 (8)	0.0014 (8)
N2	0.0360 (9)	0.0314 (10)	0.0751 (12)	−0.0035 (7)	0.0094 (8)	−0.0013 (9)
O1	0.0493 (9)	0.0342 (10)	0.0869 (11)	−0.0040 (7)	0.0124 (8)	−0.0050 (8)
Cl1	0.0650 (5)	0.0851 (6)	0.1438 (8)	−0.0346 (4)	0.0410 (5)	−0.0043 (5)

### Geometric parameters (Å, º)

**Table:**

O1W—H1WA	0.826 (16)	C6—H6A	0.9700
O1W—H1WB	0.808 (16)	C6—H6B	0.9700
C1—N1	1.466 (3)	C7—N2	1.451 (2)
C1—C2	1.516 (3)	C7—H7A	0.9700
C1—H1A	0.9700	C7—H7B	0.9700
C1—H1B	0.9700	C8—O1	1.231 (2)
C2—C3	1.514 (3)	C8—N2	1.336 (2)
C2—H2A	0.9700	C8—C9	1.493 (3)
C2—H2B	0.9700	C9—C10	1.383 (3)
C3—C4	1.515 (4)	C9—C14	1.382 (3)
C3—H3A	0.9700	C10—C11	1.381 (3)
C3—H3B	0.9700	C10—H10	0.9300
C4—C5	1.514 (3)	C11—C12	1.366 (3)
C4—H4A	0.9700	C11—H11	0.9300
C4—H4B	0.9700	C12—C13	1.372 (3)
C5—N1	1.469 (3)	C12—Cl1	1.741 (2)
C5—H5A	0.9700	C13—C14	1.383 (3)
C5—H5B	0.9700	C13—H13	0.9300
C6—N1	1.460 (3)	C14—H14	0.9300
C6—C7	1.504 (3)	N2—H2	0.8600
			
H1WA—O1W—H1WB	109 (2)	C7—C6—H6B	109.2
N1—C1—C2	111.19 (19)	H6A—C6—H6B	107.9
N1—C1—H1A	109.4	N2—C7—C6	110.77 (17)
C2—C1—H1A	109.4	N2—C7—H7A	109.5
N1—C1—H1B	109.4	C6—C7—H7A	109.5
C2—C1—H1B	109.4	N2—C7—H7B	109.5
H1A—C1—H1B	108.0	C6—C7—H7B	109.5
C3—C2—C1	111.3 (2)	H7A—C7—H7B	108.1
C3—C2—H2A	109.4	O1—C8—N2	122.74 (18)
C1—C2—H2A	109.4	O1—C8—C9	120.83 (18)
C3—C2—H2B	109.4	N2—C8—C9	116.42 (18)
C1—C2—H2B	109.4	C10—C9—C14	118.80 (18)
H2A—C2—H2B	108.0	C10—C9—C8	118.50 (18)
C2—C3—C4	109.98 (18)	C14—C9—C8	122.69 (18)
C2—C3—H3A	109.7	C11—C10—C9	120.6 (2)
C4—C3—H3A	109.7	C11—C10—H10	119.7
C2—C3—H3B	109.7	C9—C10—H10	119.7
C4—C3—H3B	109.7	C12—C11—C10	119.3 (2)
H3A—C3—H3B	108.2	C12—C11—H11	120.3
C5—C4—C3	110.9 (2)	C10—C11—H11	120.3
C5—C4—H4A	109.4	C11—C12—C13	121.5 (2)
C3—C4—H4A	109.4	C11—C12—Cl1	119.58 (18)
C5—C4—H4B	109.4	C13—C12—Cl1	118.89 (19)
C3—C4—H4B	109.4	C12—C13—C14	118.7 (2)
H4A—C4—H4B	108.0	C12—C13—H13	120.6
N1—C5—C4	111.4 (2)	C14—C13—H13	120.6
N1—C5—H5A	109.4	C9—C14—C13	120.98 (19)
C4—C5—H5A	109.4	C9—C14—H14	119.5
N1—C5—H5B	109.4	C13—C14—H14	119.5
C4—C5—H5B	109.4	C6—N1—C1	112.30 (17)
H5A—C5—H5B	108.0	C6—N1—C5	110.17 (17)
N1—C6—C7	112.24 (17)	C1—N1—C5	109.74 (16)
N1—C6—H6A	109.2	C8—N2—C7	122.38 (17)
C7—C6—H6A	109.2	C8—N2—H2	118.8
N1—C6—H6B	109.2	C7—N2—H2	118.8
			
N1—C1—C2—C3	−57.0 (3)	C11—C12—C13—C14	−1.4 (4)
C1—C2—C3—C4	53.1 (3)	Cl1—C12—C13—C14	179.04 (17)
C2—C3—C4—C5	−53.1 (3)	C10—C9—C14—C13	0.7 (3)
C3—C4—C5—N1	57.3 (3)	C8—C9—C14—C13	−178.0 (2)
N1—C6—C7—N2	163.46 (18)	C12—C13—C14—C9	0.7 (3)
O1—C8—C9—C10	28.2 (3)	C7—C6—N1—C1	69.4 (2)
N2—C8—C9—C10	−150.8 (2)	C7—C6—N1—C5	−167.94 (19)
O1—C8—C9—C14	−153.1 (2)	C2—C1—N1—C6	−177.55 (19)
N2—C8—C9—C14	27.9 (3)	C2—C1—N1—C5	59.6 (2)
C14—C9—C10—C11	−1.4 (3)	C4—C5—N1—C6	175.98 (19)
C8—C9—C10—C11	177.3 (2)	C4—C5—N1—C1	−59.9 (2)
C9—C10—C11—C12	0.7 (4)	O1—C8—N2—C7	−3.5 (3)
C10—C11—C12—C13	0.8 (4)	C9—C8—N2—C7	175.58 (18)
C10—C11—C12—Cl1	−179.72 (18)	C6—C7—N2—C8	163.4 (2)

### Hydrogen-bond geometry (Å, º)

**Table:**

*D*—H···*A*	*D*—H	H···*A*	*D*···*A*	*D*—H···*A*
O1*W*—H1*WA*···N1^i^	0.83 (2)	2.03 (2)	2.851 (3)	174 (2)
N2—H2···O1*W*	0.86	2.06	2.855 (2)	153
C6—H6*B*···O1*W*	0.97	2.59	3.406 (3)	142

Symmetry code: (i) −*x*+1/2, *y*+1/2, −*z*+1/2.
