# Supplementary figures and images for: Crystal structure of 4-chloro-N-[2-(piperidin-1-yl)eth­yl]benzamide monohydrate
Source: Acta Crystallogr E Crystallogr Commun. 2015 Jan 1;71(Pt 1):o39–40. doi: 10.1107/S2056989014026851 (PMC4331843; doi:10.1107/S2056989014026851)

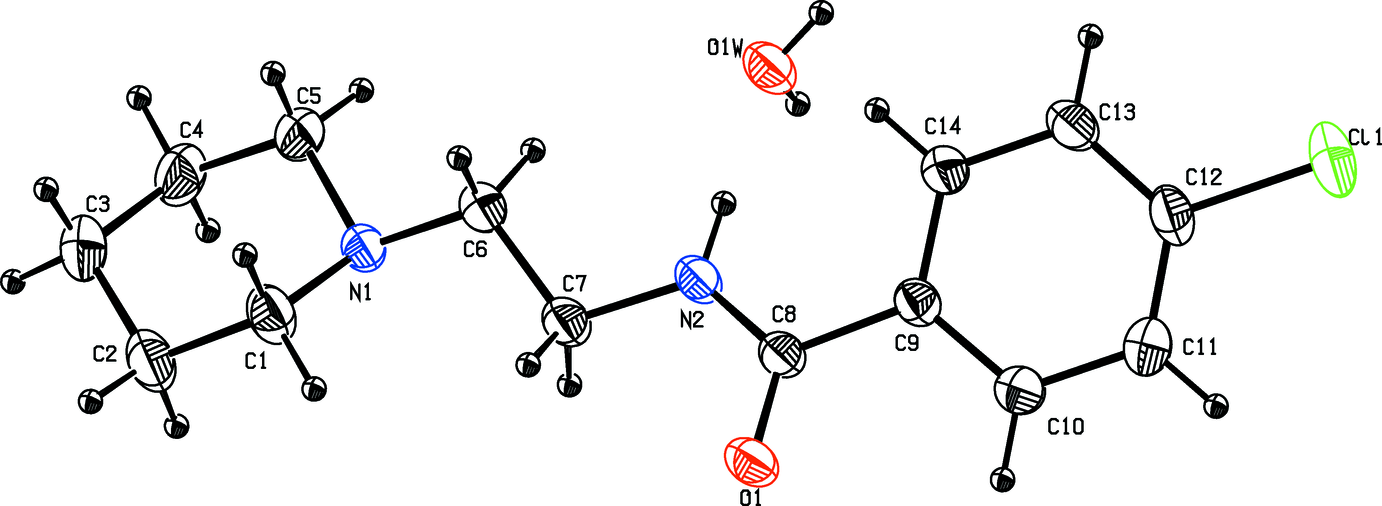

Supplement: Supplementary file 4 [file e-71-00o39-fig1.tif]

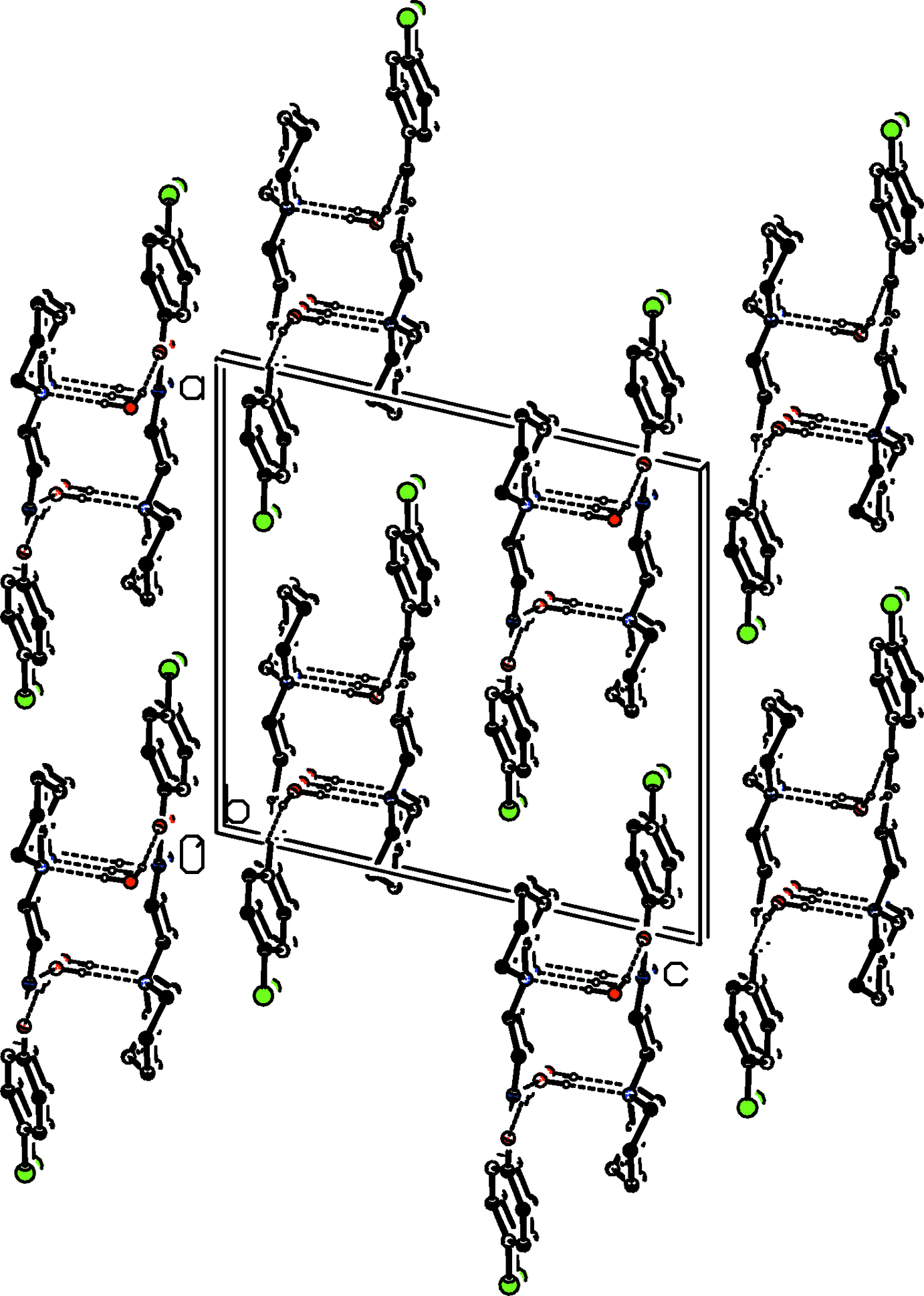

Supplement: Supplementary file 5 [file e-71-00o39-fig2.tif]
